# Complement component 1q subcomponent binding protein in the brain of the rat

**DOI:** 10.1038/s41598-019-40788-z

**Published:** 2019-03-14

**Authors:** János Barna, Diána Dimén, Gina Puska, Dávid Kovács, Vivien Csikós, Szilvia Oláh, Edina B. Udvari, Gabriella Pál, Árpád Dobolyi

**Affiliations:** 1 Department of Anatomy, Histology and Embryology, Semmelweis University, Budapest, Hungary; 2 MTA-ELTE Laboratory of Molecular and Systems Neurobiology, Department of Physiology and Neurobiology, Hungarian Academy of Sciences and Eötvös Loránd University, Budapest, Hungary; 3 Hungarian Defence Forces Military Hospital, Budapest, Hungary

## Abstract

Complement component 1q subcomponent binding protein (C1qbp) is a multifunctional protein involved in immune response, energy homeostasis of cells as a plasma membrane receptor, and a nuclear, cytoplasmic or mitochondrial protein. Recent reports suggested its neuronal function, too, possibly in axon maintenance, synaptic function, and neuroplasticity. Therefore, we addressed to identify C1qbp in the rat brain using *in situ* hybridization histochemistry and immunolabelling at light and electron microscopic level. C1qbp has a topographical distribution in the brain established by the same pattern of C1qbp mRNA-expressing and protein-containing neurons with the highest abundance in the cerebral cortex, anterodorsal thalamic nucleus, hypothalamic paraventricular (PVN) and arcuate nuclei, spinal trigeminal nucleus. Double labelling of C1qbp with the neuronal marker NeuN, with the astrocyte marker S100, and the microglia marker Iba1 demonstrated the presence of C1qbp in neurons but not in glial cells in the normal brain, while C1qbp appeared in microglia following their activation induced by focal ischemic lesion. Only restricted neurons expressed C1qbp, for example, in the PVN, magnocellular neurons selectively contained C1qbp. Further double labelling by using the mitochondria marker Idh3a antibody suggested the mitochondrial localization of C1qbp in the brain, confirmed by correlated light and electron microscopy at 3 different brain regions. Post-embedding immunoelectron microscopy also suggested uneven C1qbp content of mitochondria in different brain areas but also heterogeneity within single neurons. These data suggest a specific function of C1qbp in the brain related to mitochondria, such as the regulation of local energy supply in neuronal cells.

## Introduction

Complement component 1q subcomponent binding protein (C1qbp; UniGene code: Rn.2765) is a multifunctional and multicompartmental protein^1,2^. It was originally described and named differently based on its functions. C1qbp on the cell surface^3^ is typically referred to as a receptor of the globular head of complement component 1q (gC1qR) whose mechanisms of interaction with C1q were recently identified^4^. C1qbp (gC1qR) has been implicated in the modulation of the immune response to pathogens^5^. Different mechanisms have been proposed including the pro-inflammatory role of C1qbp (gC1qR) by promoting the migration of macrophages^6,7^, and the mediation of the actions of pro-inflammatory agents, such as high molecular weight kininogen to produce further pro-inflammatory agents^5,8^. C1qbp can also prevent cell damage by the elimination of C1q, and other ligands including antimicrobial peptides from the inflammatory site^9,10^. In addition, C1qbp (gC1qR) can serve as an entry point into the cells for several different viruses including HIV^11^, hepatitis C^12^, hantavirus causing hemorrhagic fever^13^, porcine circovirus^14^, *Staphylococcus aureus* to cause endocarditis^15^, and even *Plasmodium falciparum* infected erythrocytes causing malaria^16^. Another possible function of C1qbp in the cell surface, as hyaluronan binding protein 1 (HABP1), is its effect to bind to the major extracellular matrix protein hyaluronan. Intracellular C1qbp located in the nucleus and the cytoplasm, referred to as splicing factor-associated protein p32 (SF2-associated protein or p32) has been proposed to act as a regulator of RNA stability, which also plays a role in mRNA splicing. It can bind to an RNA binding protein, which can influence the half-life of mRNA. In relation to that, C1qbp as Y-box protein-associated acidic protein (YBAP1) may also have some transcription regulatory activity *via* its binding to Y-box proteins^17^. C1qbp (YBAP1) inhibits the interaction between Y-box protein 1 (YB-1) and transportin 1 in the cytosol preventing YB-1 function in the nucleus as a transcription factor but promoting its action in the cytosol as a component of the messenger ribonucleoprotein particle (mRNP), thus, C1qbp may act as an mRNP remodelling protein^18^. Cytosolic C1qbp can translocate into the nucleus upon mitogenic stimulation and phosphorylation by the MAP kinase^19^. However, C1qbp is located in most cell types in the mitochondria^20–22^ where it is responsible to maintain transcription of mitochondrial proteins and their functioning^23^, thereby contributing to a sufficiently high level of oxidative phosphorylation^24^, and may even protect against oxidative stress^25^. Indeed, patients with mutations in C1qbp demonstrate cardiomyopathy associated with respiratory chain deficiencies^26^. Mice, lacking C1qbp are not viable and die at early embryonic age^27^. In turn, mice with selective loss of C1qbp in cardiomyocytes showed contractile dysfunction, enlarged cardiac mitochondria and died earlier (at 14 months) than control mice^28^. The fibroblasts of C1qbp knock-out mice show multiple defects in oxidative phosphorylation^27^, which can be restored with the expression of C1qbp in them^26^. In contrast, overexpression of C1qbp leads to apoptosis in fibroblasts and other non-tumour cells^29–31^ possibly by the generation of excess reactive oxygen species^32^, but rather supports the survival of cancer cells by contributing to extra energy production for growth^33^.

C1qbp was found to be overexpressed in several different types of tumours^28,34,35^, its expression level inversely correlated with the prognosis of the patients^36–38^, and a genetic polymorphism increased breast cancer risk^39^. A proposed potential mechanism is that C1qbp receptor gets solubilized^40^ so that tumour cells can evade from destruction by complement^41^. Mitochondrial C1qbp may also be responsible for the tumour-promoting property by increasing the energy for the growth of the cells and allowing glutamine addiction^42^. In addition, C1qbp may also contribute to metastasis^37^, for example, insulin-like growth factor induced the transition of C1qbp to the plasma membrane in pancreatic cancer cells, which promoted hepatic metastasis^43^. Inhibition of C1qbp was reported to suppress the growth of the tumour cells, which presents C1qbp as a potential drug target^34,43,44^. In addition, cell surface C1qbp was also investigated for targeted tumour drug delivery^45^. Furthermore, a small molecule able to penetrate through the blood-brain barrier, and into mitochondria, was recently developed with potential future application in gliomas^46^.

Despite the intriguingly diverse functions of C1qbp in different organs of the body, our knowledge on C1qbp is much more limited in the central nervous system, which contains the highest numbers of different cell populations. C1qbp has been reported to be expressed in the cerebral cortex and the cerebellum^21^. We recently found using proteomics that C1qbp has higher level in synaptosomal than cell body mitochondria suggesting uneven distribution of the protein in neurons^47^. C1qbp level may also change in relation to physiological state of the brain as we found its reduced level in hypothalamic synaptosomes in mother as compared to non-maternal rats^48^. While the importance of this change has not been elucidated, it has been reported that cytosolic C1qbp can interact with the intracellular loop of different G-protein coupled receptors including beta subunits of GABA_A_ receptors^49^, vasopressin^50^ and adrenergic receptors^51,52^ and alter their ligand binding properties^50^ or subcellular localization^51^. Acting *via* these receptors or by altering energy availability for brain cell compartments, are potential mechanisms how C1qbp can affect information transmission in the central nervous system. Recently, a mouse line, in which C1qbp is selectively absent in the central nervous system was developed. These mice showed leukoencephalopathy, demyelination and axon loss in some midbrain and brainstem regions^53^. The oligodendrocytes were damaged, vacuoles were formed and astrocytosis took place in the affected brain regions. While neurological symptoms were not present in the forebrain, epileptic seizures occurred in some of the conditional knock-out animals suggesting forebrain abnormalities as well^53^. Therefore, it is a major gap in our knowledge base that the distribution of C1qbp in the central nervous system has not been reported previously. It is also unknown if glial and different neuronal cell types express C1qbp and what the cellular localization of this protein is in the different cell types. To address these questions, we mapped C1qbp in the brain by using *in situ* hybridization histochemistry as well as immunohistochemistry and compared the distribution of C1qbp mRNA and protein. Double immunolabelling with neuronal and glial markers was also performed to identify the cell types, which express C1qbp in the brain. In addition to the normal brain, C1qbp immunoreactivity was also examined following hypoxic lesion produced by occlusion of the middle cerebral artery^54^. Furthermore, pre- and post-embedding immunoelectron microscopy and double fluorescent labelling with the mitochondrial marker isocitrate dehydrogenase subunit alpha (Idh3a) was applied to determine the subcellular localization of C1qbp in some neurons containing a substantial amount of the protein.

## Results

### Expression pattern of C1qbp mRNA

**Figure 1:**
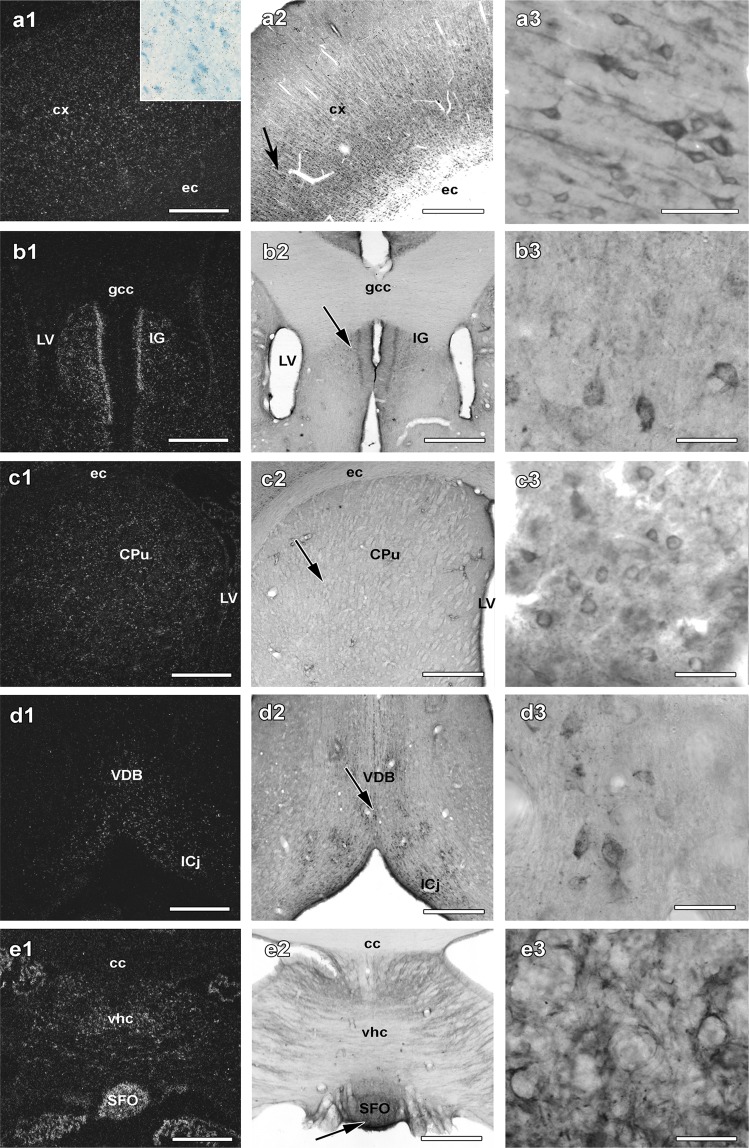
C1qbp in the cortical and subcortical forebrain. Dark-field images demonstrate the appearance of C1qbp mRNA in the cortical (**a1**) and subcortical (**b1**,**c1**,**d1** and **e1**) forebrain. The inserted panel of a1 shows a high magnification bright field image of the cortical region labelled for C1qbp mRNA also by *in situ* hybridization histochemistry to demonstrate autoradiography grains as black dots distributed above labelled cells. The same fields in the middle panels show the distribution of C1qbp immunoreactivity resembles to that of C1qbp mRNA (**a2**,**b2**,**c2**,**d2** and **e2**). High magnification images of the cortical and subcortical regions are pointed to by the arrows in the middle panels are demonstrated in the right panels (**a3**,**b3**,**c3**,**d3** and **e3**). C1qbp is abundant in the cerebral cortex (**a1** and **a2**) but not in the white matter of the corpus callosum (**b1** and **b2**), the indusium griseum (IG in **b1** and **b2**), in the caudate putamen (CPu in **c1** and **c2**), in the ventral limb of the diagonal band of Broca (VDB in **d1** and **d2**) and in the subfornical organ (SFO in **e1** and **e2**). Additional abbreviations: cx – cerebral cortex, ec – external capsule, gcc – genu of corpus callosum, ICj – island of Calleja, LV – lateral ventricle, vhc – ventral hippocampal commissure. Scale bars = 1 mm for (**a1**,**a2**,**b1**,**b2**,**c1**,**c2**,**d1**,**d2**,**e1** and **e2**) 40 μm for (**a3**,**b3**,**c3**,**d3** and **e3**).

The distribution of C1qbp mRNA was the same using 2 different non-overlapping probes when adjacent sections were labelled. C1qbp mRNA was relatively widespread in the brain but it was not found in the white matter. Within the grey matter, C1qbp was present in a variety of different brain regions but absent in some other parts of the brain (Table 1). C1qbp-expressing neurons were relatively evenly distributed in different parts of the cerebral cortex but showed some layer-specificity. Their density was the highest in layer 5 while they were absent in layer 1 (Fig. 1a1). In addition, we analysed the percentage of cells expressing C1qbp in the cerebral cortex in layer 5, which was (4 or more autoradiography grains above the cell) 58.3%. The average grain number of C1qbp-expressing cells was 8.8. Furthermore, *in situ* hybridization histochemistry images at high magnification (inserted panel of Fig. 1a1) showed that even in brain areas where C1qbp is abundant, there are cells, which do not express C1qbp as we demonstrate it also in the cortex. The septum contained a relatively high density of labelled cells in its medial part while only a low number of labelled cells were present in its lateral part.

**Table 1:** Comparison of the distribution of C1qbp mRNA expression with C1qbp immunoreactivity in the rat brain. The number of plus symbols (+) in the column “mRNA level” is proportional to the density of cell bodies expressing C1qbp mRNA based on *in situ* hybridization histochemistry in the area. A cell was considered to express C1qbp if at least 4 autoradiography grains were above the Giemsa-labelled cell as the number of autoradiography grain in the same cell-free area was less than 2. The number of plus symbols (+) in the other columns are proportional to the density of C1qbp-ir cell bodies and fibres, respectively, in the given brain region.

Area	mRNA level	C1qbp-ir cell bodies	C1qbp-ir fibres
**Forebrain Cerebral cortex**			
Layer I	0	0	+++
Layer II	+	0	+++
Layer III	++	+	++++
Layer IV	+	+	++++
Layer V	+++	+++	++++
Layer VI	+	++	++++
**Septum**			
Medial septal nucleus	++	++	+++
Lateral septum intermed. nucleus	+	+	+
Lateral septum ventral nucleus	+	0	+++
Indusium griseum	+++	++++	+
Nucleus of the vertical limb of the diagonal band of Broca	+++	+++	+
Subfornical organ	++++	+++	++++
Vascular organ of lamina terminalis	+++	+++	++
**Choroid plexus**	++	++	++
**Basal nuclei**			
Caudate-Putamen	+++	++	+
Globus pallidus	+	+	+++
Endopiriform nucleus	++	++	+
Claustrum	+	+	0
Nucleus accumbens	+++	+++	+
Ventral pallidum	+++	+++	+
Substantia innominata	+	+	++
**Diencephalon**			
Thalamus			
Anteromedial nucleus	++	++	+
Anterodorsal nucleus	++++	+++++	++
Anteroventral nucleus	+++	++	+
Midline and intralaminar nuclei	+++	++	++
Lateral nuclei	+	+	+
Ventral nuclei	+++	+++	++
Reticular nucleus	+	+	+
Medial habenular nuclei	++++	++++	++
Lateral habenular nuclei	+	+	0
Posterior nuclei	+	+	++
**Hypothalamus**			
Medial preoptic area	+++	++++	++
Lateral preoptic area	++++	+++++	++
Supraoptic nucleus	+++	++++	+++
Suprachiasmatic nucleus	+	0	+
Anterior hypothalamic area	+	0	++
Paraventricular nucleus	++++	+++++	++
Arcuate nucleus	+++	+++	+++
Lateral hypothalamic area	+++	+	++
Ventromedial nucleus	+	+	++++
Dorsomedial nucleus	+	0	+
Posterior hypothalamic area	++	++	+++
Mamillary body	+	+	+
**Dorsal Hippocampus**			
CA1 region	++	++	++
CA2 region	++	+++	+++
CA3 region	+++	+	++
Dentate gyrus	+++	+++	++++
**Amygdala**			
Central nucleus	++	++	++++
Basal nuclei	++	++	+++
Lateral nucleus	+	0	++
Medial nucleus	+	0	+++
Cortical nucleus	+	0	+++
**Hindbrain Pons**			
Pontine nuclei	++	++	+
Superior olive	++	+	++++
Inferior olive	0	0	+
Nucleus of the trapezoid body	+++	+++	++++
Pontine reticular formation	++	+	+++++
Sensory trigeminal nu.	+++	+++	++++
Pontine raphe pallidus nuclei	+++	++++	+
Superior vestibular nucleus	++	+++	+++++
Medial vestibular nucleus	++	+++	+++++
Facial motor nucleus	+++	++	+++
**Cerebellum**			
Cortex			
Molecular layer	0	0	++
Purkinje cell layer	0	0	+
Granule cell layer	++++	++	++++
Deep cerebellar nuclei	+++	++	0
**Medulla Oblongata**			
Cochlear Nuclei	++	++	+++
Gigantocellular reticular nucleus	++	++	+++
Spinal trigeminal nucleus	+	+	+++++
Medullary reticular formation	0	0	++++
Inferior olive	0	0	+

A high number of labelled cells were present in the indusium griseum (Fig. 1b1), in the caudate putamen (Fig. 1c1) and also ventrally to it in the ventral limb of the diagonal band of Broca (Fig. 1d1). In turn, C1qbp mRNA was abundant in the accumbens nucleus and the ventral pallidum while other basal nuclei, such as the globus pallidus, the claustrum and the substantia innominate contained only few C1qbp-expressing cells.

While the plexus choroideus was only moderately labelled, the circumventricular organs including the subfornical organ (Fig. 1e1) and the vascular organ of the lamina terminalis contained a very high level of C1qbp mRNA.

**Figure 2:**
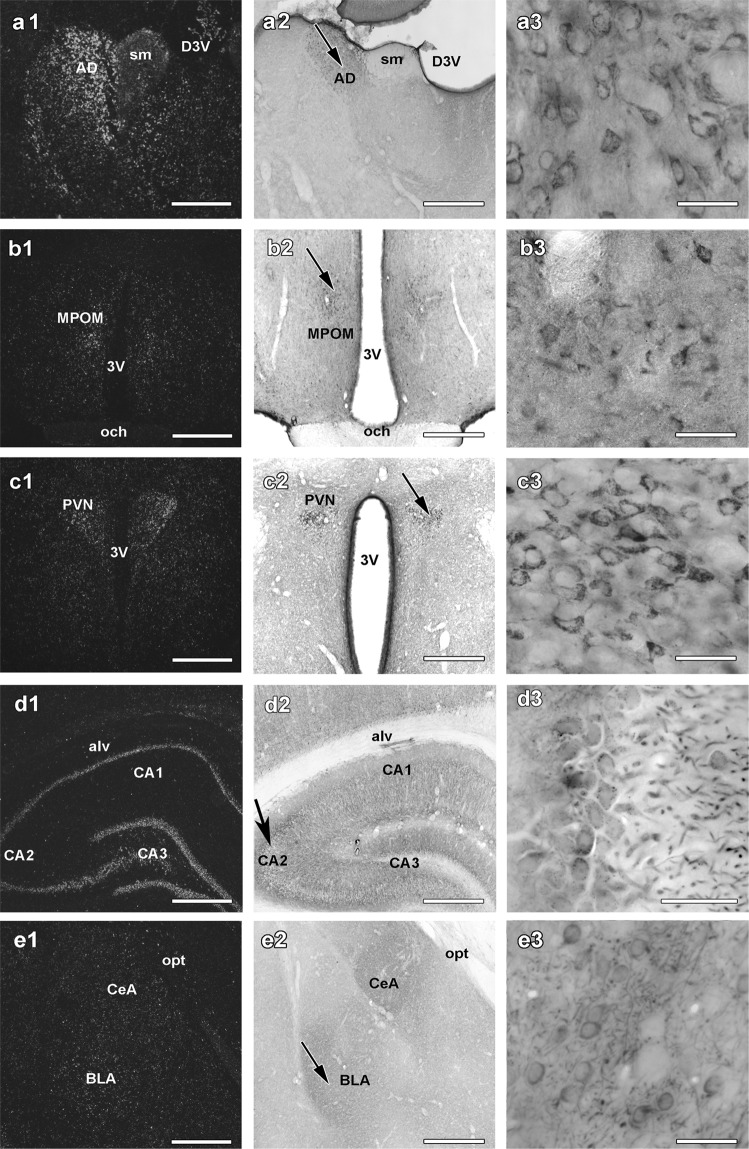
C1qbp in the diencephalon, hippocampus and amygdala. Distribution of cells that express mRNA encoding C1qbp in the diencephalon (**a1**,**b1**,**c1**), in the hippocampus (**d1**) and in the amygdala (**e1**). The same fields are shown following C1qbp immunolabelling (**a2**,**b2**,**c2**,**d2** and **e2**). The arrows mark the magnified part of the examined regions which are demonstrated in the right panels (**a3**,**b3**,**c3**,**d3** and **e3**). C1qbp is abundant in the anterodorsal thalamic nucleus (AD in a1 and **a2**), the medial preoptic area (MPOM in **b1** and **b2**), the paraventricular hypothalamic nucleus (PVN in **c1** and **c2**), the CA1-3 regions and the dentate gyrus of the hippocampus (CA1, CA2 and CA3 in **d1** and **d2**), in the central and basolateral (CeA and BLA in **e1** and **e2**) amygdaloid nuclei. Additional abbreviations: 3 V – 3rd ventricle, alv – alveus, D3V – dorsal 3rd ventricle, och - optic chiasm, opt – optic tract, sm – stria medullaris. Scale bars = 1 mm for (**a1**,**a2**,**b1**,**b2**,**c1**,**c2**,**d1**,**d2**,**e1** and **e2**) 40 μm for (**a3**,**b3**,**c3**,**d3** and **e3**).

In the diencephalon, C1qbp had a topographical distribution too. A particularly abundant expression was present in the medial habenula and the anterodorsal thalamic nucleus (Fig. 2a1). The density of C1qbp-expressing neurons was also high in other anterior nuclei, the midline and intralaminar thalamic nuclei as well as the ventral thalamic nuclei. In turn, only few labelled cells were observed in the lateral habenula, the lateral and posterior thalamic nuclei as well as in the reticular thalamic nucleus. There were also hypothalamic nuclei observed with high level of C1qbp mRNA. In the anterior hypothalamus, the preoptic area contained a high density of C1qbp cells in the medial preoptic nucleus (Fig. 2b1) as well as in the magnocellular preoptic nucleus. C1qbp mRNA was also abundant in the supraoptic and paraventricular thalamic nuclei (Fig. 2c1) while other parts of the anterior thalamus were mostly devoid of C1qbp expression. In the mediobasal region, the arcuate nucleus contained the highest number of C1qbp-expressing neurons. A considerable amount of labelled cells was also present in the lateral and posterior hypothalamic areas. In contrast, the ventromedial and dorsomedial nuclei, as well as the mammillary body contained only a low level of C1qbp-expressing cells.

In the hippocampus, C1qbp-expressing cells were present in all regions, albeit at a higher density in the dentate gyrus and the CA2 region. The granule cell layer and the pyramidal cell layer were the most intensively labelled, respectively (Fig. 2d1). In the amygdala, C1qbp-expressing cells had the highest density in the central and basal nuclei (Fig. 2e1). Other parts of the amygdala showed only a moderate number of labelled cells.

**Figure 3:**
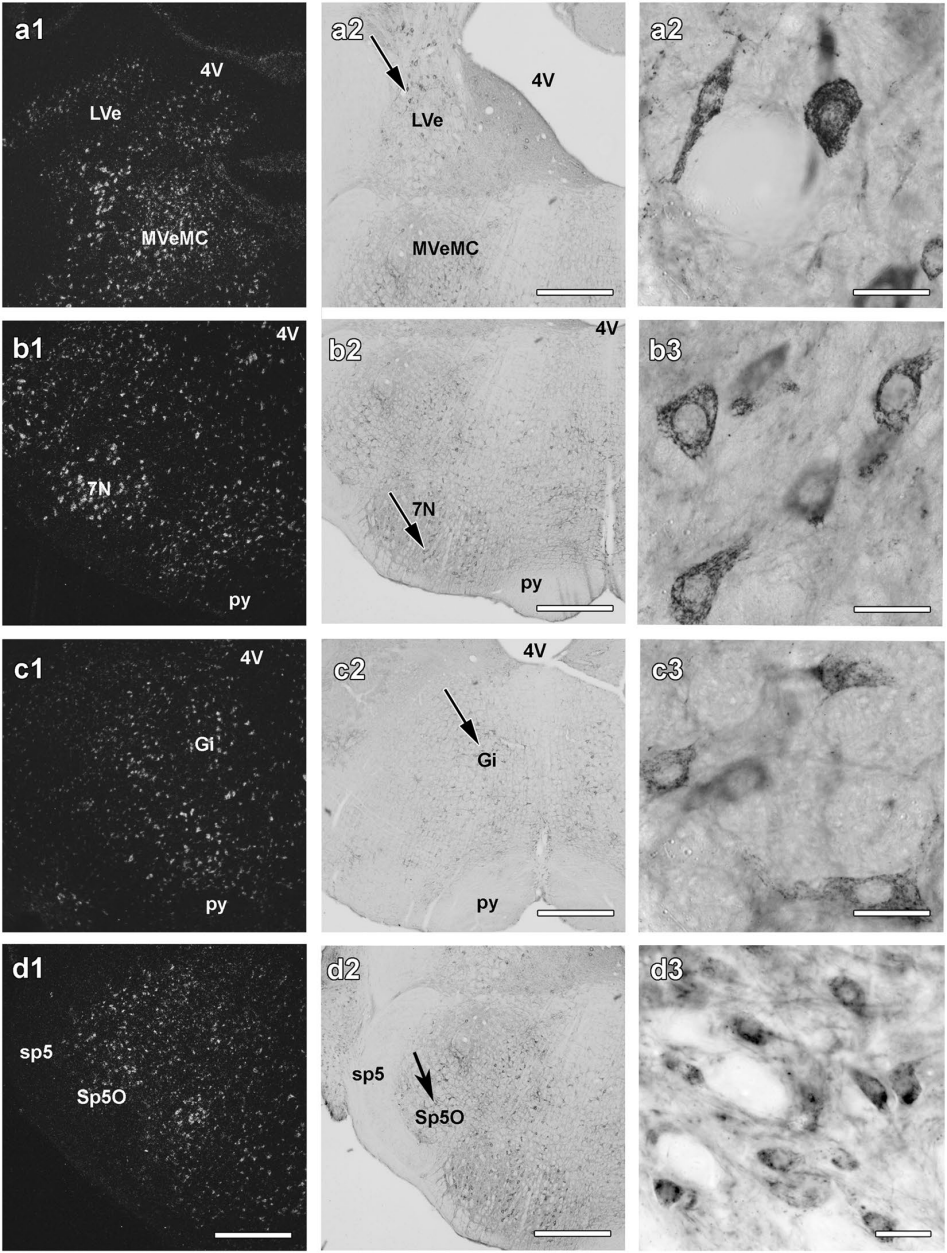
C1qbp in the brainstem. Dark-field images of C1qbp *in situ* hybridization histochemistry sections of the brainstem are shown in the left panels (**a1**,**b1**,**c1** and **d1**). The very same fields are shown following C1qbp immunohistochemistry in the middle panels (**a2**,**b2**,**c2** and **d2**). The right panels (**a3**,**b3**,**c3** and **d3**) show higher magnification images from the brain areas pointed to by the arrows in the middle panels. C1qbp is abundant in the magnocellular part of the medial vestibular nucleus (MVeMC in **a1** and **a2**) and the lateral vestibular nucleus (LVe in **a1** and **a2**), the facial motor nucleus (7N in **b1** and **b2**), the gigantocellular nucleus (Gi in **c1** and **c2**) and in the Sp5O sensory core of the trigeminal nucleus (Sp5O in **d1** and **d2**). Additional abbreviations: 4V – 4th ventricle, py -pyramidal tract, sp5- spinal trigeminal tact. Scale bars = 1 mm for (**a1**,**a2**,**b1**,**b2**,**c1**,**c2**,**d1** and **d2**) 40 μm for (**a3**,**b3**,**c3** and **d3**).

In the midbrain, pons, and medulla oblongata, only few structures exhibited a high level of C1qbp expression. These include the vestibular nuclei (Fig. 3a1), the pontine raphe nucleus, the facial motor nucleus (Fig. 3b1), the reticular formation particularly the gigantocellular region (Fig. 3c1), the nucleus of the trapezoid body, and the sensory trigeminal nucleus (Fig. 3d1). A moderate density of C1qbp was also found in the pontine nuclei, the superior olive, and the cochlear nuclei. In the cerebellum, the granule cell layer and the deep nuclei contained a high density of C1qbp-expressing cells while C1qbp mRNA was absent in the molecular and Purkinje cell layers of the cerebellum.

### Distribution of C1qbp immunoreactivity

C1qbp-positive structures were present in several brain regions. Typically, cell bodies contained the most intense labelling but examination of the material at higher magnification revealed that dendrites and thin fibres also contain C1qbp (middle and right panels of Figs 1, 2 and 3). The topographical distribution of C1qbp-immunoreactive (C1qbp-ir) cell bodies was very similar to the distribution of C1qbp mRNA (Table 1). The few differences include some brain areas, such as the amygdaloid nuclei, the ventral subdivision of the lateral septal nucleus, the anterior hypothalamic area, the dorsomedial hypothalamic nucleus and the granule cell layer of the cerebellum where a higher density of C1qbp-expressing cells was found as compared to the number of C1qbp-ir cells. In fact, the subregional distribution of C1qbp mRNA and immunoreactivity also correlated remarkably well, which was demonstrated in the subcortical forebrain (Fig. 1 panels 2–3 of a, b, c, d, e), in the diencephalon (Fig. 2 panels 2–3 of a, b, c, d, e;) as well as in the brainstem (Fig. 3 panels 2–3 of a, b, c and d). In these figures note the similarities between the middle immunohistochemistry panels and the left *in situ* hybridization panels. In the few brain regions, where C1qbp mRNA but not immunolabelled cells were found, C1qbp-ir fibres were present. The density of C1qbp-ir fibres resembled to that of the cell bodies. Labelled fibres were always abundant in brain areas where labelled cell bodies were described. Some of them were thick processes while often thin fibres were also observed. In addition, a high intensity of C1qbp-ir fibre network was present in some brain regions where labelled cell bodies were scarce, including the superficial layers of the cerebral cortex, several amygdaloid nuclei, the ventral subdivision of the lateral septum, the globus pallidus, the ventromedial hypothalamic nucleus, the superior olive, the reticular formation, and the molecular layer of the cerebellum.

### Neuronal presence of C1qbp

**Figure 4:**
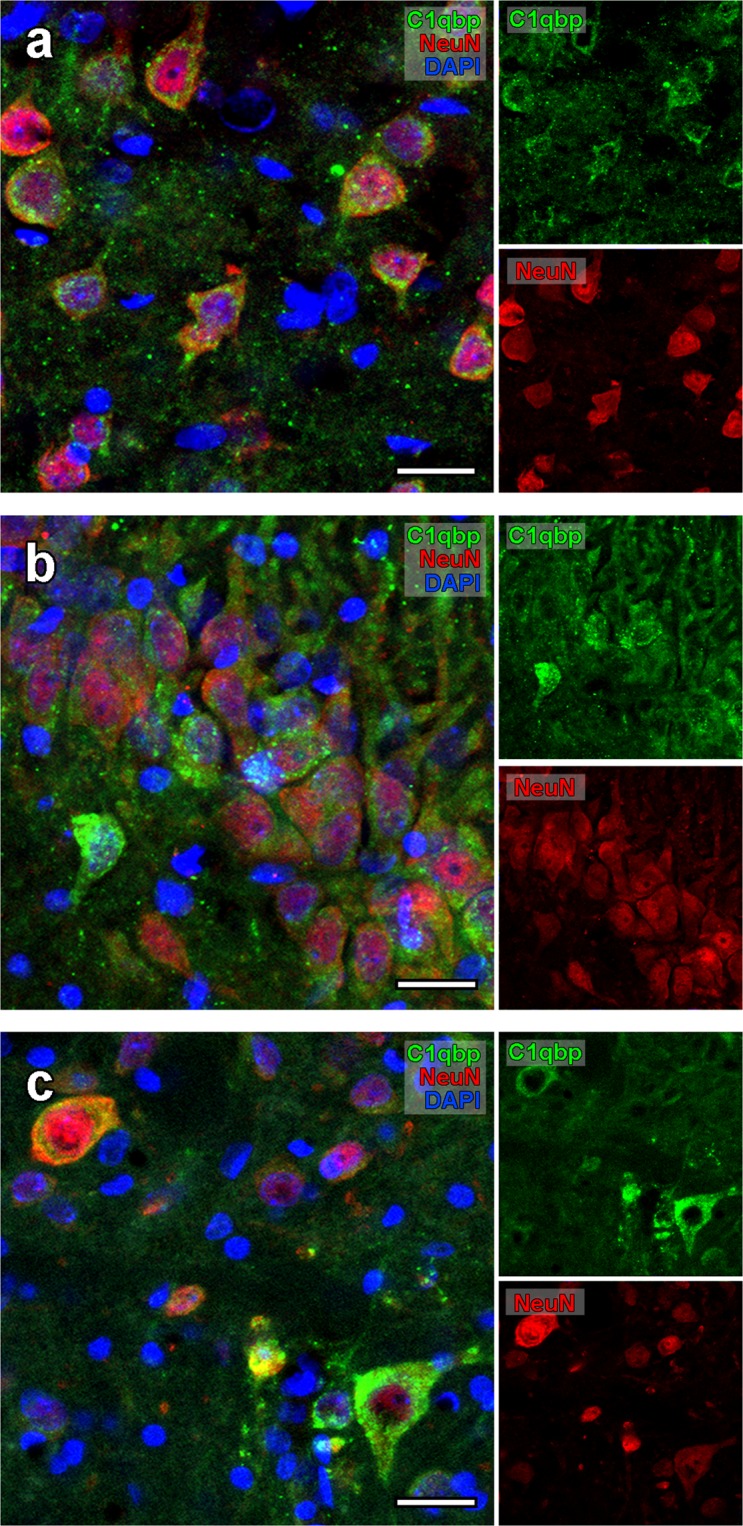
Neuronal localization of C1qbp in the cortex, hippocampus and spinal trigeminal nucleus showed by fluorescent microscopy. The confocal images demonstrate double labelling of C1qbp (green) and NeuN (red) in the cortex (**a**), in the CA2 region of the hippocampus (**b**) and in the spinal trigeminal nucleus (Sp5O), the sensory core of the brainstem (**c**). The nuclei are labelled by DAPI. Scale bars = 10 μm for (**a**–**c**).

Based on the high magnification light microscopic findings (Figs 1a3, b3, c3, d3 and e3, 2a3, b3, c3, d3 and e3 and 3a3, b3, c3 and d3) the morphology of C1qbp labelled cells suggested that neurons are labelled in all brain regions where C1qbp was present. In order to certify our suggestion, we made fluorescent double labelling with the neuronal marker NeuN, which confirmed the neuronal expression of C1qbp in the cortex (Fig. 4a), in the CA2 region of the hippocampus (Fig. 4b) and in the spinal trigeminal nucleus (Fig. 4c). The topographical distribution of C1qbp in the brain and its absence in some brain areas, as well as its restricted localization within some nuclei suggested that C1qbp is not expressed in all neurons. The results of fluorescent double labelling underlying the selective presence of C1qbp in the neuronal cells.

### Co-localization studies of C1qbp in the brain

**Figure 5:**
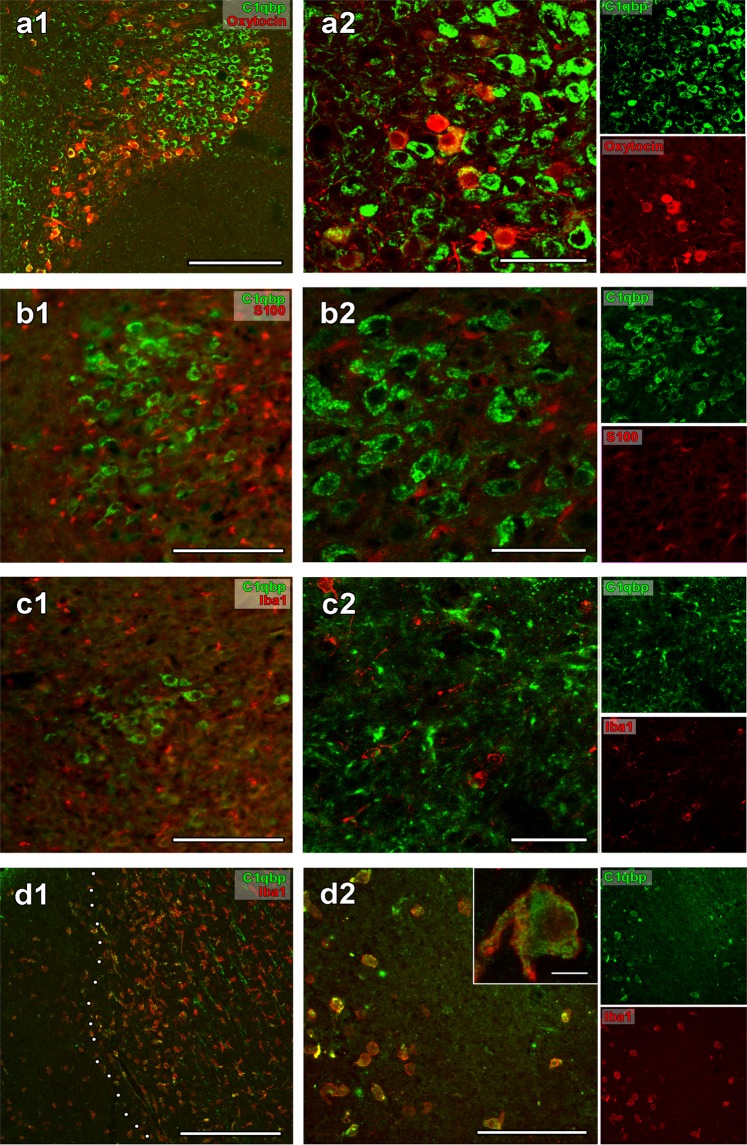
Co-localization of C1qbp with oxytocin and glial markers. Double labelling of C1qbp (green) and oxytocin (red) (**a1**) in the PVN. High magnification demonstrates the co-localization between the markers (**a2**). Double labelling of C1qbp (green) and astrocyte marker S100 (red) (b1), and C1qbp (green) and microglia marker Iba3 (red) (**c1**). The high magnification confocal images show the lack of co-localisation between C1qbp and glial cell markers (S100 in b2, Iba3 in **c2**). C1qbp-ir cells within the lesioned area in the cortex at 72 h following a focal ischemic attack (**d**). Low magnification panel of the lesioned brain double labelled with C1qbp (green) and Iba1 (red) (**d1**). The border of the lesion is indicated by white dots. Higher magnification image of a double labelled section indicated that C1qbp (green) co-localizes with Iba1 (red) in the infarct area (**d2**). The inserted panel of high magnification confocal image demonstrates C1qbp in dot-like structures within an Iba1-positive cell. Scale bars = 500 μm for a1 and d1; 300 μm for (**b1** and **c1**) 100 μm for a2,b2,c2 and d2; 10 μm for inserted panel of d2.

The above described neuronal appearance of C1qbp showed that there are neuronal cells, which do not contain C1qbp. Addressing this question, we demonstrated that C1qbp is present in essentially all oxytocin neurons (Fig. 5a1 and a2) in the PVN. The localization and morphology of C1qbp-positive but oxytocin-negative neurons suggested that they are also magnocellular neurons but most other neurons inside and immediately outside the PVN do not contain C1qbp (Fig. 5a1 and a2).

Based on the results of double fluorescent labelling with neuronal marker we concluded that all the C1qbp-positive cells were labelled with NeuN. However, NeuN-negative cells were typically not labelled suggesting that glial cells may not contain C1qbp. This finding was confirmed by double labelling of C1qbp with the astrocyte marker S100 (Fig. 5b1 and b2) and the microglia marker Iba1 (Fig. 5c1 and c2) in the PVN. We could not detect any astrocyte or microglia, which contained C1qbp in the physiological brain.

We were interested in whether the activation of microglial cells result in the appearance of C1qbp in these cells. Following focal ischemia, the above described pattern of C1qbp immunoreactivity looked unaffected in the cortex and striatum on the intact side of the brain as well away from the ischemic lesion ipsilaterally. In contrast, a novel labelling pattern of C1qbp appeared within the infarct area. Some cells were strongly labelled with C1qbp, which co-localized with Iba1 within the infarct area as well as the penumbra 72 h after ischemia (Fig. 5d1 and d2). Their distribution was uneven. However, they were present generally all over in the infarct area. In addition, C1qbp-containing cells also appeared around the lesion in all cerebral layers as well as in the caudate putamen (striatum).

### Subcellular distribution of C1qbp

**Figure 6:**
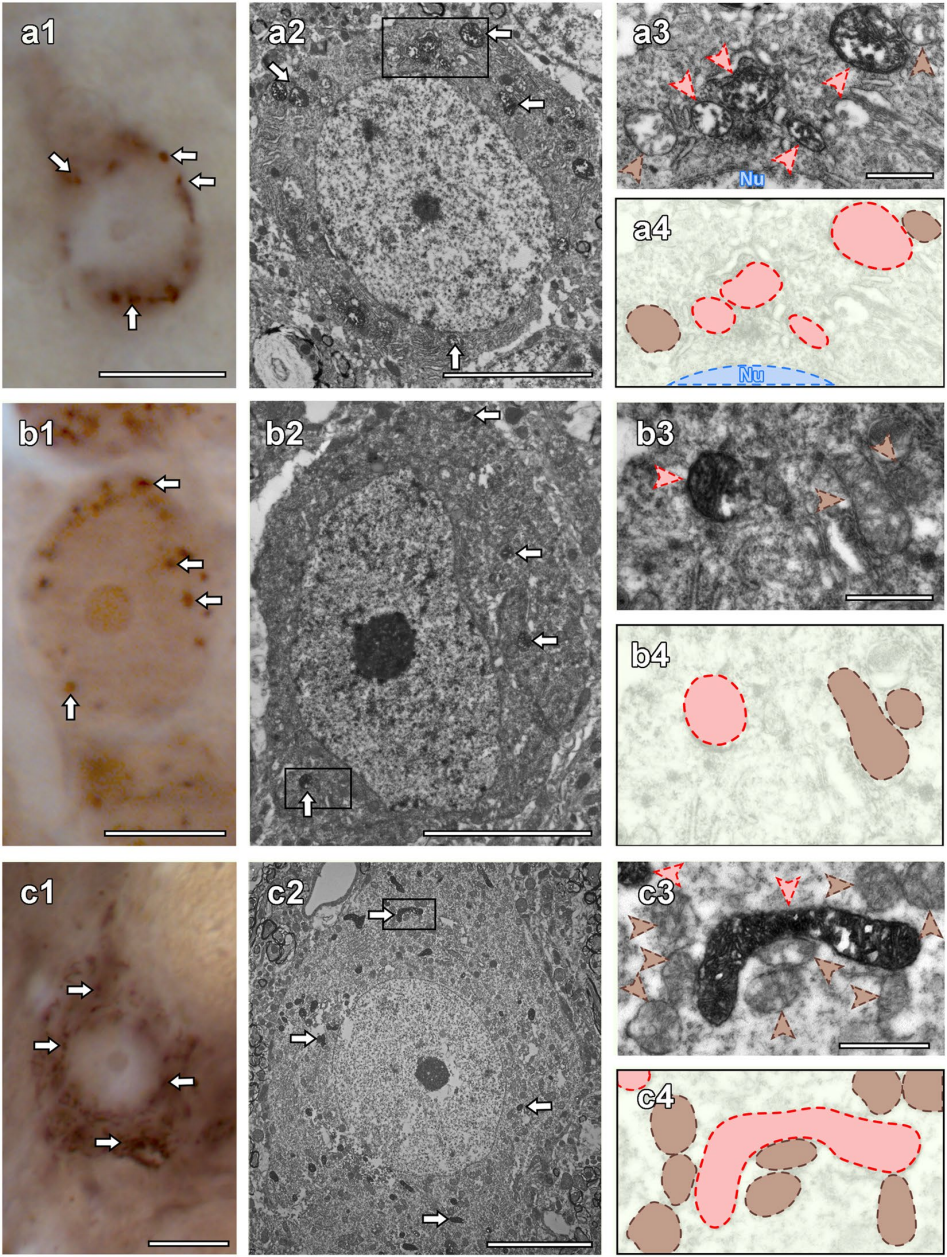
Mitochondrial localization of C1qbp in the cortex, hippocampus and spinal trigeminal nucleus showed by electron microscopy. Light micrographs of C1qbp immunolabelling show pronounced C1qbp positive dot-like staining of a cortical neuron (**a1**), a CA2 neuron (**b1**) and a sensory neuron of the spinal trigeminal nucleus (Sp5O) in the brainstem (**c1**). Correlated electron micrographs of the same neurons (**a2**,**b2**,**c2**). The white arrows are pointed C1qbp-ir mitochondria that are also marked by white arrows in panel (a1, b1 and c1). The mitochondria in the framed areas in panel (a2, b2 and c2) are shown in high magnification (**a3**,**b3**,**c3**) and those schematic illustrations (**a4**,**b4**,**c4**) to demonstrate C1qbp-positive (red arrowheads, red coloured shape) and C1qbp-negative (brown arrowheads, brown coloured shape) mitochondria in the examined neurons. Abbreviation: Nu – nucleus. Scale bars = 10 μm for (**a1**,**b1** and **c1**) 5 μm for (**a2**,**b2** and **c2**) 500 nm for (**a3**,**b3**, and **c3**).

The correlative light and electron microscopic approach revealed the subcellular localization pattern of C1qbp in different brain areas. Based on the results of *in situ* hybridization histochemistry and light microscopic labelling, cells in layer 5 of the frontal cortex were first addressed at the ultrastructural level. We found that pyramidal cells contained dot-like, elliptic-shaped, or sometimes irregularly elongated C1qbp immunolabelling (Fig. 6a1). After analysing the electron micrograph of the examined cell showed by light microscope, the labelled structures were defined as mitochondria (Fig. 6a2 and a3). As strong C1qbp labelling was found in the CA regions of the hippocampus, we also determined cellular localization of C1qbp in the CA2 region as well. Light (Fig. 6b1) and electron microscopic (Fig. 6b2, and b3) examination of the same CA2 neuron revealed the predominantly mitochondrial localization of C1qbp immunoreactivity in the hippocampus. Comparison of the light microscopic labelling with its electron microscopic counterparts confirmed a mitochondrial localization of C1qbp immunoreactivity in a large neuron of the spinal trigeminal nucleus, too (Fig. 6c1, c2 and c3).

### Comparison of the subcellular localization pattern of C1qbp in different brain areas

**Figure 7:**
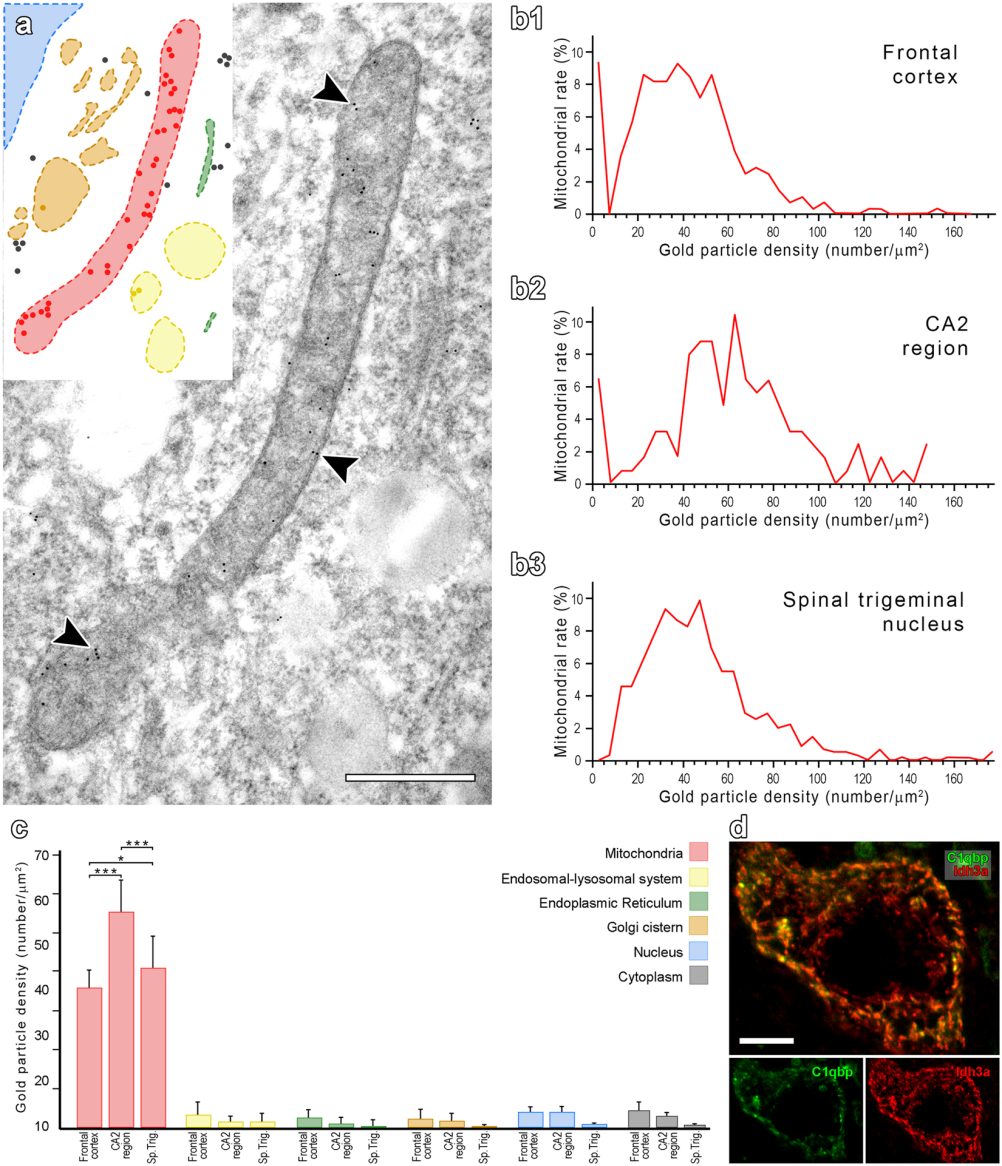
Comparison of the subcellular localization pattern of C1qbp between distinct cellular compartments in different brain regions. C1qbp immunolabelling shows different compartmental distribution of the protein (**a**). Some of the 10 nm gold particles relating to C1qbp immunoreactivity are pointed by arrowheads. Coloured drawing insertion is demonstrated for better understanding. Colour coding: red-mitochondria, yellow-endosomal-lysosomal system, green-endoplasmic reticulum, orange-Golgi cistern, blue-nucleus, non-coloured-cytoplasm. Darker coloured and black dots are equivalent to gold particles. The histograms of mitochondria based on C1qbp immunoreactivity related immunogold densities show differences between individual mitochondria in the pyramidal neurons of the frontal cortex (**b1**), CA2 neurons of the hippocampus (**b2**), sensory neurons of the spinal trigeminal nucleus (**b3**). Comparative results of the analysis of mean C1qbp-related immunogold particle densities in the three examined cell groups (**c**). Horizontal lines above bars indicate that p value is significant (*0.01 < p < 0.05; **0.001 < p < 0.01; ***p < 0.001). Mean values with standard error indicate pooled data of immunogold particle densities in distinct compartments from all cells of the examined brain areas. The magnified confocal image shows double labelling of C1qbp (green) and the mitochondrial marker Idh3a (red) (**d**). Abbreviation: Sp. Trig. – spinal trigeminal nucleus. Scale bars = 500 nm for a; 10 μm for (**d**).

We demonstrated mitochondrial presence of C1qbp in neurons of the frontal cortex, dorsal hippocampus and the sensory part of the spinal trigeminal nucleus by post-embedding immunoelectron microscopy, too. We found strong C1qbp immunolabelling in mitochondria compared to other cellular organelles (Fig. 7a). First, we were interested in the distribution pattern of C1qbp between the different cellular compartments and between distinct brain areas. We examined the C1qbp related immunogold particle area densities (hereinafter referred shortly to C1qbp density) calculated from the number of the immunogold particles per µm2 in different cellular compartments (mitochondria, endosomal-lysosomal system, Golgi cistern, endoplasmic reticulum, nucleus and cytoplasm). On the one hand, we examined the mitochondrial C1qbp distribution pattern demonstrated by histograms based on mitochondrial immunogold particle densities of three examined neuron groups: the pyramidal cells of the frontal cortex (Fig. 7b1), CA2 cells of the dorsal hippocampus (Fig. 7b2) and sensory neurons of the spinal trigeminal nucleus (Fig. 7b3). The frequency distribution of the histograms showed that there may be different mitochondrial populations based on C1qbp densities. A significant ratio of mitochondria does not contain C1qbp immunoreactivity in the pyramidal cells of the frontal cortex and hippocampal CA2 cells. Linear logistic regression model was used to evaluate the effect of mitochondrial size due to the sectioning of the three-dimensional shape of the organelle. There is no relation between the mitochondrial size and C1qbp density in these cell groups. On the other hand, two-way analysis of variance test revealed significant differences between the different cell organelles in each examined neuron group. Moreover, comparing the compartmental distribution of the three brain areas with each other also showed significant differences. *Post-hoc* Tukey test revealed significantly higher C1qbp density in mitochondria than in other six cellular organelles. Furthermore, the C1qbp density was higher in mitochondria of the CA2 region when compared to neuronal mitochondria in pyramidal cells of the cerebral cortex and neurons of the sensory trigeminal nucleus (p < 0.001 for both comparisons). The mitochondrial C1qbp density also differed significantly (p < 0.05) between the spinal trigeminal sensory neurons and cortical pyramidal cells but there were no differences between the other compartments in each brain region (Fig. 7c).

## Discussion

The brain distribution of C1qbp will first be discussed including the specificity of labelling at the mRNA and protein level. Then, the selective neuronal and mitochondrial localization of C1qbp will be interpreted in relation to previous literature in the field. Finally, potential functional consequences of the findings will be summarized.

### Distribution of C1qbp in the brain

The probes necessary for *in situ* hybridization histochemistry were prepared from brain cDNA already suggesting that C1qbp is expressed in the central nervous system. Two non-overlapping probes were used for *in situ* hybridization histochemistry, which provided the same distributional pattern using adjacent sections confirming the specificity of the labelling. Using these probes, the initial RT-PCR finding of C1qbp expression in the brain was confirmed by *in situ* hybridization histochemistry, which in turn also revealed a topographic distribution. Radioactive *in situ* hybridization histochemistry is a very sensitive technique allowing the determination of cells with even low expression level of C1qbp. Using this method, only relatively few brain regions containing grey matter were completely devoid of C1qbp in accordance with previous western blotting brain localization studies^23^. Since the labelling intensity of individual cells is proportional to their expression level, it was possible to perform a semi-quantitative determination of C1qbp expression in the brain. These determined brain nuclei with particularly high level of C1qbp expression. These areas were not confined to a specific brain region and did not belong to a single functional network.

### Neuronal-specific expression of C1qbp

The specificity of C1qbp immunolabelling has been suggested by our previous Western blotting data^48^ as well as Western blotting (coupled with siRNA-based downregulation of the C1qbp) validation of others using the same anti-C1qbp antibody as what used in the present study^55–57^. In addition, in the present study we provided further strong evidence of the specificity of C1qbp antibody, because when a similar distribution of C1qbp mRNA-expressing and C1qbp-containing cell bodies were detected throughout the rat brain. The similarity was observed even at a subregional scale within restricted brain regions. There were, however, few brain regions with detected expression of mRNA without C1qbp-ir cell bodies. We believe that the explanation for these few discrepancies is the lower detection limit using radioactive *in situ* hybridization than immunolabelling.

High magnification *in situ* hybridization histochemistry indicated that only a portion of cells are labelled even in brain regions expressing C1qbp mRNA at a high density, such as the cerebral cortex, where 41.7% of the cells were negative for C1qbp mRNA. This labelling pattern held for immunolabelling as well. Using this technique, however, double labelling was also possible to conduct, which revealed that the C1qbp-ir cells are all neurons in the normal brain as they were labelled with the neuronal marker NeuN. Consistent with this finding, we did not find any co-localization between C1qbp and the astrocyte marker S100 and the microglia marker Iba1 suggesting that neither glial cell type contain C1qbp. In sharp contrast to the neuronally restricted localization of C1qbp in the normal brain, it appeared in microglia activated by ischemic lesion both within the infarct area and in the penumbra surrounding it suggesting the capability of microglia expressing C1qbp under specific conditions.

C1qbp immunoreactivity was not only present in cell bodies but also in fibres. Some of these fibres were thick dendrites among labelled cell bodies. In addition, a fine fibre network was visible, too, even in some brain regions where C1qbp-ir cell bodies were not observed. The morphology of these fibres suggests that they are axons. Their appearance in brain region without C1qbp-ir cell bodies may indicate a remote origin of these projections.

### C1qbp as a selective mitochondrial protein in the brain

Analysis of high magnification light microscopic images revealed that C1qbp immunoreactivity is present in dot-, elliptic-, and sometimes irregularly elongated line-like intracellular structures reminiscent of mitochondria in neurons and also in activated microglia. Furthermore, an essentially complete co-localization was found between C1qbp and the mitochondrial marker isocitrate dehydrogenase subunit alpha (Idh3a), an enzyme of the citric acid cycle. Correlated light and electron microscopy also confirmed the mitochondrial localization of C1qbp immunoreactivity. These data were verified in neurons located in 3 different brain regions, the cerebral cortex, the hippocampus, and the spinal trigeminal nucleus. Since light microscopy did not indicate C1qbp in the plasma membrane at any part of the brain, we conclude that C1qbp is likely absent in the plasma membrane of neuronal cells. Additional post-embedding immunogold labelling was performed in the selected 3 brain regions to determine whether other cell organelles contain C1qbp. A very low level of C1qbp was detected in a variety of different organelles, the density of C1qbp in the mitochondria is at least 10 times more than in any other organelles suggesting a predominantly mitochondrial localization of C1qbp in neurons. These data support previous findings reporting that C1qbp was predominantly present in the mitochondrial fraction of homogenized and fractionated brain tissue^23^. This restricted localization of C1qbp in neurons is in contrast to several other cell types in the periphery, such as immune cells, where C1qbp was detected in the plasma membrane, the nucleus and also in the cytoplasm^3,5,20,21^.

### Proposed functions of C1qbp in the brain

Based on the mitochondrial distribution of C1qbp in neurons and activated microglia, a mitochondrial function of the protein can be concluded. Previous studies in peripheral cell types suggested its role in the regulation of oxidative phosphorylation, and also the regulation of mitochondrial gene expression either as a chaperone or as a transcription factor^17,24,32^. Among the many previously suggested functions of C1qbp, these mitochondrial functions but not immunosignalling of surface C1qbp^6,8,58^ and cytosolic/nuclear mRNA chaperone functions^17,18^ are likely based on its subcellular distribution in brain cells.

What makes C1qbp unique among mitochondrial proteins is that it is not ubiquitously present in all mitochondria. As discussed above, mitochondria of glial cells can function without C1qbp in them. C1qbp is not the first protein found in mitochondria of neurons but not in astrocytes, e.g. the mitochondrial phosphocreatine/creatine kinase was also found to be absent in glial cells^59^ and proteomics data suggest several other differentially expressed proteins^60^. The absence of C1qbp in astrocytes could be related to their less intense oxidative phosphorylation, as astrocytes can rely on glycolysis for ATP^61^. Furthermore, the fact that some grey matter brain regions do not express C1qbp suggests that certain neuronal cell types also are without C1qbp in their mitochondria. The mitochondrial density of C1qbp of CA2 pyramidal cells was higher than in other investigated regions suggesting that C1qbp level may be different even between C1qbp-expressing neurons. Furthermore, double labelling with the mitochondrial marker Idh3a suggested that mitochondria in the same neuron may contain different levels of C1qbp as the ratios of intensities of the 2 labellings differed in distinct mitochondria. Consistent with this finding, the histogram of mitochondria based on the density of C1qbp immunogold labelling using post-embedding electron microscopy suggested that a significant ratio of mitochondria do not contain C1qbp. Thus, C1qbp may be functional only in a portion of the mitochondria even within the same cell. The heterogeneity of mitochondria within the same cell is not often investigated as most methods does not have the resolution required for investigating individual mitochondria. When such techniques are applied, however, differences between individual mitochondria are not uncommon^62–64^. The individual characteristics of mitochondria could be related to their different status e.g. aging, activity, or the local energy demand. In fact, it was recently hypothesized that neuronal mitochondria can be divided to low activity, so called stem mitochondria, which can avoid DNA damage for future divisions due to low free radical formation, a consequence of active oxidative phosphorylation, and high activity so called differentiated mitochondria supplying ATP for the cells^65^. In addition to some mitochondria in the cell body, synaptic mitochondria were suggested to be particularly active^66^. We previously found using proteomics approaches that synaptic mitochondria contain increased amount of C1qbp^47^. Thus, it is possible that C1qbp appears selectively in high-activity mitochondria. This hypothesis is in line with the previously proposed protective function of C1qbp against reactive oxygen species^67^ and also with its ability to increase oxidative phosphorylation^68^, e.g. by increasing the activity of the pyruvate dehydrogenase complex *via* direct interaction to one of its components^24^. In addition to contributing to the maintenance of elevated oxidative phosphorylation, C1qbp may also be involved in the elimination of overused, and therefore, potentially damaged mitochondria as it was shown to be required for mitophagy^55^. As an example of physiological regulation of C1qbp content, we showed that it increases in hypothalamic neuronal terminals as rats become mothers^48^ suggesting that C1qbp content of the mitochondria can be regulated based on demand. A similar regulatory mechanism could also play a role in microglia where C1qbp appeared in mitochondria only following activation of the cells as microglial activation is known to increase the energy demand of this cell type, too^69^. Although the mechanism how C1qbp is targeted to selected mitochondria is not known, its increased cellular expression level may be regulated by the transcription factor ZNF32^25^.

### Future directions: potential significance of C1qbp in brain diseases

Mitochondrial abnormalities have been implicated in several neurodegenerative diseases including Alzheimer’s disease, sclerosis multiplex, Parkinson’s disease, etc. C1qbp has been shown to be involved with the E3 ubiquitin ligase named parkin to potentially affect mitochondrial dysfunction in Parkinson’s disease^23^. Mice lacking C1qbp in the central nervous system showed white matter degeneration accompanied by progressive oligodendrocyte loss and axon degeneration^53^. C1qbp, as a regulatory and protective mitochondrial protein might be involved in a variety of different neuropathologies, which are to be examined in future studies.

## Methods

### Animals

All animal experimentations were approved by the Animal Examination Ethical Council of the Animal Protection Advisory Board at the Eötvös Loránd University, Budapest, and Semmelweis University, Budapest, and met the guidelines of the Animal Hygiene and Food Control Department, Ministry of Agriculture, Hungary. A total of 20 male Wistar rats (250–350 g adult body weight; Charles Rivers Laboratories, Budapest, Hungary) were used in this study: 6 rats for *in situ* hybridization, 8 rats for light microscopic examination, 2 rats for electron microscopic analyses, and additional 4 male rats for middle cerebral artery occlusion. Animals were kept on standard laboratory conditions with 12-h light, 12-h dark periods (lights on at 06:30) and supplied with dry rat food and drinking water *ad libitum*. Rats were housed three per cage at a temperature of 22 ± 1 °C before experiments. The animals were deeply anesthetized by an intraperitoneal injection of a mixture of 20% urethane and distilled water (1.0 g/kg body weight) before decapitation or cardiac perfusion.

### Middle cerebral artery occlusion

Focal ischemia was induced using a modified intraluminal suture method of the described previously^70^. Briefly, left common, internal and external carotid arteries were exposed through a midline neck incision and were carefully dissected from the surrounding tissues under an operating microscope. After electrocoagulation of the external and common carotid arteries, a 3–0 silicon rubber-coated monofilament (Doccol, Redlands, CA) was inserted through the common carotid artery into the internal carotid artery 18 to 20 mm beyond the carotid bifurcation to the base of the middle cerebral artery. The pterygopalatine branch of the internal carotid artery was exposed before the insertion in order to avoid the filament turning into it. An atraumatic aneurysm clip (Codman, Johnson and Johnson, Le Locle, NE, Switzerland) was placed on the internal carotid artery to prevent bleeding. The clip and the monofilament were removed 1 h later, and the incision was sutured. The rats were sacrificed 72 h after the beginning of the reperfusion and transcardially perfused as described below.

### Production of *in situ* hybridization probe for C1qbp

Preparation of the *in situ* hybridization probes was performed as described previously^71^. First, RT-PCR was carried out using total RNA isolated from frozen rat brain. The concentration of RNA was adjusted to 2 µg/µl, and it was treated with Amplification Grade DNase I (Invitrogen). Then, cDNA was synthesized using SuperscriptII (Invitrogen) as suggested in the kit protocol. The cDNA was subsequently diluted (10x), and 2.5 µl of the resulting cDNA was used as template in PCR reactions iTaq DNA polymerase (Bio-Rad Laboratories, Hercules, CA, USA). The rat C1qbp cDNA sequence (NCBI Reference Sequence: NM_019259.2) was PCR amplified using the following primer pairs: A: GGGCCTTGTATGACCACCTA and TGATGTCAAGGCAGCTTTTG, B: TAGCATCCCTCCAACCTTTG and TCCCTCCACTCAGAGTCACC. The PCR products were purified from gel, inserted into TOPO TA cloning vectors (Life Technologies) and transformed chemically into competent bacteria. Selected plasmids were applied as templates in PCR reactions, using the above described primer pairs specific for C1qbp, with the reverse primers also containing a T7 RNA polymerase recognition site. At the end, the identities of the cDNA probes were verified by sequencing.

### *In situ* hybridization histochemistry

To describe the expression pattern of C1qbp in the brain, the fresh tissue was quickly frozen on dry ice. *In situ* hybridization histochemistry was processed as described previously^72^. Briefly, serial coronal sections (12 µm) were cut using a cryostat from bregma level +3.5 mm to −15 mm, mounted on positively charged slides (SuperfrostUltraPlus™; Thermo Fisher Scientific, Pittsburgh, PA, USA), dried, and stored at −80 °C until use. Antisense [35 S]UTP-labelled riboprobes were generated using T7 RNA polymerase of the MAXIscript *in vitro* transcription kit (Ambion, Austin, TX) from PCR-amplified fragments of the cDNA subcloned into TOPO TA vectors.

Tissue was prepared using an mRNA-locator Kit (Ambion) according to manufacturer’s instructions. For hybridization, we used 80 µl hybridization buffer and 1 million DPM of labelled probe per slide. Washing procedures included a 30 min incubation in RNase A, followed by decreasing concentrations of sodium-citrate buffer (pH = 7.4) at room temperature, and then at 65 °C. After drying, slides were dipped in NTB nuclear track emulsion (Eastman Kodak, Rochester, NY), stored for 3 weeks at 4 °C for autoradiography. Then, the slides were developed and fixed with Kodak Dektol developer and Kodak fixer, respectively, counterstained with Giemsa, dehydrated, and coverslipped with Cytoseal 60 (Stephens Scientific).

### Tissue collection for light and electron microscopy

Rats were deeply anesthetized with urethane (1.0 g/kg body weight), which was followed by transcardial perfusion with 4% paraformaldehyde in 0.1 M PB (pH = 7.4) for light microscopic immunolabelling and with the following mixture for correlative light and electron microscopy: 0.05% glutaraldehyde, 15% saturated picric acid, 4% paraformaldehyde in 0.1 M PB (pH = 7.4). After the perfusion, the brains were immersion fixed in the same solutions for 12 h at room temperature and then washed thoroughly with 0.1 M PB. Subsequently, the brains were saturated with 20% sucrose in 0.1 M PB. On a sliding microtome, 50 µm thin serial coronal sections of the rat brains were sectioned between 3.5 and −15.0 mm bregma levels. The sections were collected in PB containing 0.05% sodium azide and stored at 4 °C until further processing.

### C1qbp immunohistochemistry

Every fourth free-floating section of 5 rats was immunolabelled for C1qbp using a rabbit anti-C1qbp antibody (dilution 1:50, sc-48795, Santa Cruz Biotechnology Inc.). Western blotting using this antibody revealed a single band from cellular homogenates^55–57^. In addition, downregulation of C1qbp expression using C1qbp-specific siRNA resulted in a reduced density band corresponding to C1qbp in Western blotting^56,57^.

The immunolabeling was performed as described before^48^. Briefly, endogenous peroxidase activity was blocked with 0.9% H_2_O_2_. Non-specific binding sites were blocked with 3% bovine serum albumin (BSA), 0.5% Triton-X 100 and 0.05% sodium azide dissolved in PB for 1 h. After washing steps, the sections were incubated in biotin conjugated goat anti-rabbit immunoglobulin G (IgG) secondary antibody (1:1000, Jackson Immunoresearch, West Grove, PA) for 1 h and followed by the applying of avidin-biotin peroxidase complex (ABC; Vector Laboratories, Burlingame, CA, USA) for 1 hour. The labelling was visualized by incubation in the mixture of 0.02% 3,3-diaminobenzidine (DAB; Sigma), 0.08% nickel (II) sulphate, and 0.001% H_2_O_2_. After dehydration, sections were mounted on SuperFrost Ultra Plus Adhesive Slides coverslipped with DPX Mounting Medium.

### Fluorescent double labelling of C1qbp

Every fourth free-floating coronal brain section of 3 rats was immunolabelled for C1qbp as described above except that the visualization of C1qbp was performed using FITC-tyramide (1:8000) amplification followed by incubation in the following antibodies: rabbit anti-Idh3a as a marker of mitochondria (1:40; Proteintech, cat number: 15909-1-AP), mouse anti-oxytocin (1:1000; Abcam, Cambridge, UK, cat number: ab78364), mouse anti-NeuN as a marker of neuronal nuclei (1:500; Millipore, Billerica, MA, cat. number: MAB377), mouse anti-S-100, as a marker of astrocytes (1:2500 Sigma-Aldrich, cat. number: S2532), rabbit anti-ionized calcium-binding adapter molecule 1 (Iba1) as a marker of microglial cells (1:1000; Wako, cat. number: 019–197419). Following application of the primary antiserum, sections were incubated in donkey Alexa Fluor 594-conjugated anti-mouse or anti-rabbit secondary antibody (1:400, Life Technologies, Grand Island, NY) for 2 h. Subsequently, the sections were mounted on slides and coverslipped in antifade medium (Prolong Antifade Kit, Molecular Probes).

### Light microscopy and image processing

Sections were examined using an Olympus BX60 light microscope equipped with fluorescent epi-illumination and a dark-field condenser. Images were captured at 2048 × 2048 pixel resolution with a SPOT Xplorer digital CCD camera (Diagnostic Instruments, Sterling Heights, MI) using 4-60x objectives. Confocal images were acquired with a Zeiss LSM 780 Confocal Microscope System using 20-60x objectives at an optical thickness of 1–3 µm. There were 6 z-stack images taken for each area. Images were adjusted using the “levels” and “sharpness” commands in Adobe Photoshop CS 8.0. Full resolution of the images was maintained until the final versions, which were adjusted to a resolution of 300 dpi.

The number of autoradiography grains for quantification purposes was calculated on bright-field images of sections labelled with *in situ* hybridization histochemistry obtained using 100x objectives. A grain (black dot) was included to a cell if it was located above the blue Giemsa-stained cell body.

### Pre-embedding electron microscopic immunostaining

Free-floating brain sections including the frontal cortex, dorsal hippocampus and the sensory part of the spinal trigeminal nucleus were used for correlative light and electron microscopy. After 1% sodium borohydride pretreatment, sections were rinsed extensively in 0.05 M Tris buffer (pH 7.6) and then endogenous peroxidase activity was blocked with 0.9% H_2_O_2_ and non-specific binding sites were blocked with 10% fetal calf serum, 5% BSA dissolved in the same buffer. Sections were incubated with anti-C1qbp antibody at a dilution of 1:60 for 48 h at room temperature then biotin conjugated goat anti-rabbit IgG secondary antibody at 1:500 was applied for 24 h. Followed by the incubations for 6 h with ABC reagent (Vector Laboratories, Burlingame, CA, USA), the immunoreactions were revealed by nickel-enhanced 3,3-diaminobenzidine (Ni-DAB; DAB peroxidase substrate kit, SK-4100, Vector Laboratories, Burlingame, CA, USA). Then the sections were post-fixed in 0.5% OsO_4_ for 30 min and stained with 1% aqueous uranyl acetate for 1 h. Samples were embedded in Durcupan resin (ACM, Sigma) following dehydration by a graded series of ethanol and acetonitrile. Samples were embedded on slides and polymerized for 48 h at 60 °C. After light microscopic observations, we reembedded the corresponding pieces of the selected brain areas containing C1qbp-immunopositive cells and sectioned by a Reichert Jung ultramicrotome. Sections were collected onto formvar coated slot copper grids and counterstained by lead citrate for 30 s prior to ultrastructural investigation.

### Low temperature embedding for electron microscopic immunogold labelling

The same brain areas as used for pre-embedding electron microscopic examinations were prepared for post-embedding immunocytochemistry. Samples were rinsing thoroughly in 0.05 M maleate buffer (pH = 5.5) to remove precipitate forming ions and stained *en bloc* with 2% uranyl acetate dissolved in the same buffer for 2 h. Then the samples were fully dehydrated by placing them into a graded series of ethanol meanwhile the temperature was decreased to −20 °C. It was followed by the infiltration with pure LR White (Sigma) for 24 h at −20 °C. After 12 h, the resin was replaced for fresh one twice. Then, the specimens were put into LR White resin containing 2% benzoyl peroxide. Gelatine capsuled samples were polymerized by a DL-103 12 W ultraviolet lamp for 48 h at −20 °C. Finally, 70–80 nm thin sections were prepared by ultramicrotome and were collected onto 300 mesh nickel grids.

### Post-embedding electron microscopic immunolabelling

One series of immunostaining was performed on the same day on all sections of the cortical pyramidal layer, hippocampal CA2 region and the sensory part of the trigeminal nucleus which was ensured by toluidine blue stained semi-thin sections. Two girds were used for C1qbp immunolabelling procedure and other one grid was applied for no first antibody control in every case of the different brain regions. After treatment with 3% H_2_O_2_ for 2 min, 1% sodium borohydride and 50–50 mM glycine-ammonium chloride dissolved in 0.05 M Tris buffer (pH = 7.6) containing 0.9% sodium chloride (TBS) was used to retrieve antigens. TBS was also used for all washing steps and antibody dilutions. Non-specific binding sites were blocked with 5% BSA dissolved in TBS. Sections were incubated with anti-C1qbp antibody at a dilution of 1:20 containing 0.5% BSA and 0.05% sodium azide for 24 h at room temperature. Then, we used 10 nm gold-conjugated goat anti-rabbit secondary antibody (G7402, Sigma) for visualisation at a dilution of 1:50 in 1% BSA-TBS for 6 h. After washing three times with TBS, sections were post-fixed with 2% glutaraldehyde in TBS for 10 min. Then, washing with double distilled water three times more, the grids were air drying. The sections were stained with 1% aqueous uranyl acetate for 20 min followed by lead citrate for 30 sec.

### Electron microscopic image acquisition and processing

Electron micrographs were taken by a side-mounted Morada CCD camera (Olympus Soft Imaging Solutions) connected to a JEOL 1011 electron microscope operating at 60 kV. Images were adjusted when necessary using Adobe Photoshop CS6 (Adobe Systems).

### Morphometric analysis of C1qbp immunoreactivity

We used cell based morphometric evaluation to examine the neuronal subcellular distribution pattern of C1qbp in different brain areas. Three neuronal populations showing C1qbp immunoreactivity were examined in the study: pyramidal cells of the frontal cortex, CA2 cells of the hippocampus and sensory neurons of the spinal trigeminal nucleus. We randomly selected 10 neurons (5-5 neurons of two male rats respectively) of two grids (5-5 neurons per grid) in all cell groups (in total 30 neurons). The analyses were performed using ultrathin sections stained by C1qbp antibody and the nanogold conjugated detection system enabled the quantification.

For image analysis, greyscale images at 20 x magnification were captured using a Jeol JEM 1011 electron microscope operating at 60 kV. For the evaluation of C1qbp immunoreactivity, in each analysed brain areas, 10 cells were cut out and their different cellular compartments were identified using Adobe Photoshop CS6: cytoplasm, nucleus, endoplasmic reticulum, endosomal-lysosomal system, Golgi cistern and mitochondria. Then, we marked the immunogold particles related to the examined cell organelles. Black and white images were prepared separately of the selected areas and gold particle markings for further processing. We converted the images to 8-bit and used the software ImageJ, version 1.50i (Wayne Rasband, National Institute of Health, USA) for further analyses. On the one hand, we measured the compartmental areas in µm2, using the scale bar of the microscope software. On the other hand, we measured the number of the immunogold particles inside the six cell compartments. Then, we calculated the C1qbp related nanogold particle densities and defined it as the level of C1qbp immunoreactivity. Moreover, the values were corrected by using control sections with the omission of first antibody during the immunolabelling procedure to eliminate false positive signal of non-specific background related to our secondary antibody.

### Statistical analysis

We performed statistical analyses using the statistical package IBM SPSS Statistics Version 17. Two-way analysis of variance test was used to establish the significance between the immunogold particle density values of C1qbp immunopositivity. A Tukey test was used for *post-hoc* comparisons. We analysed differences between cellular compartments of neurons pooled from the same brain areas. Moreover, we analysed whether C1qbp labelled immunogold particle densities differ between the distinct brain areas on the cellular compartment level. We tested the effect of the mitochondrial size differences in the three-dimensional shape of the organelle after sectioning by using linear logistic regression model. We made histogram analysis to explore whether there are different mitochondrial populations within the cells in the relation of C1qbp immunoreactivity
